# Supplementary material for: Neutrophil-to-Lymphocyte Ratio and Platelet-to-Lymphocyte Ratio as Prognostic Markers for Advanced Non-Small-Cell Lung Cancer Treated with Immunotherapy: A Systematic Review and Meta-Analysis
Source: Medicina (Kaunas). 2022 Aug 8;58(8):1069. doi: 10.3390/medicina58081069 (PMC9413376; doi:10.3390/medicina58081069)
Supplement: Supplementary file 1 [file medicina-58-01069-s001.zip › Supplementary File S2_Risk of bias.pdf]

Supplementary file S2

Studies	Selection	Comparability	Outcome	Total
Katayama et al.	☆☆☆☆	☆☆	☆-☆	8 – Good
Suh et al.	☆☆☆☆	☆☆	☆☆☆	9 – Good
Khunger et al.	☆☆☆☆	☆☆	☆☆☆	9 – Good
Nakaya et al.	☆☆-☆	☆☆	--☆	6 – Moderate
Russo et al.	☆☆☆☆	☆☆	☆-☆	8 – Good
Svaton et al.	☆☆☆☆	-☆	☆-☆	7 – Good
Takeda et al.	☆☆☆☆	-☆	☆-☆	7 – Good
Zer et al.	☆☆☆☆	-☆	☆☆☆	8 – Good
Pavan et al.	☆☆☆☆	-☆	☆-☆	7 – Good
Matsubara et al.	☆☆☆☆	☆☆	☆-☆	8 – Good
Prelaj et al.	☆☆☆☆	-☆	☆-☆	7 – Good
Russo et al.	☆☆☆☆	☆☆	☆-☆	8 – Good
Zer et al.	☆☆☆☆	☆☆	☆-☆	8 – Good
Yang et al	☆☆☆☆	-☆	☆-☆	7 – Good
Ksienski et al	☆☆☆☆	-☆	☆☆☆	8 – Good
